# In vitro study of chlorine dioxide on porcine intestinal epithelial cell gene markers

**DOI:** 10.1002/vms3.658

**Published:** 2021-10-20

**Authors:** Orsolya Palócz, Zoltán Noszticzius, Kristóf Kály‐Kullai, Emma Bradley, György Csikó

**Affiliations:** ^1^ Department of Pharmacology and Toxicology University of Veterinary Medicine Budapest Hungary; ^2^ Department of Physics, Faculty of Natural Sciences Budapest University of Technology and Economics Budapest Hungary

**Keywords:** chlorine dioxide, cytochrome genes, inflammatory markers, intestinal cells

## Abstract

**Background:**

Chlorine dioxide (ClO_2_) is an inorganic, potent biocide and is available in highly purified aqueous solution. It can be administered as an oral antiseptic in this form.

**Objectives:**

Our aim is to determine the level of inflammatory markers and cytochrome genes expressed by enterocytes exposed to different concentrations of hyperpure chlorine dioxide solution.

**Methods:**

Porcine jejunal enterocyte cell (IPEC‐J2) cultures were treated with the aqueous solution of hyper‐pure chlorine dioxide of various concentrations. We determined the alterations in mRNA levels of inflammatory mediators, such as *IL6, CXCL8/IL8*, *TNF*, *HSPA6 (Hsp70)*, *CAT* and *PTGS2 (COX2)*; furthermore, the expression of three cytochrome genes (*CYP1A1*, *CYP1A2*, *CYP3A29*) were analysed by quantitative PCR method.

**Results:**

The highest applied ClO_2_ concentration reduced the expression of all three investigated CYP genes. The gene expression of *PTGS2* and *CAT* were not altered by most concentrations of ClO_2_. The expression of *IL8* gene was reduced by all applied concentrations of ClO_2_. *TNF* mRNA level was also decreased by most ClO_2_ concentrations used.

**Conclusions:**

Different concentrations of chlorine dioxide exhibited immunomodulatory activity and caused altered transcription of CYP450 genes in porcine enterocytes. Further studies are needed to determine the appropriate ClO_2_ concentration for oral use in animals.

## 1 INTRODUCTION

Chlorine dioxide (ClO_2_) is a transfer‐oxidising agent with high efficacy and speed in killing pathogens, vegetative bacteria, spores, viruses and fungi. ClO_2_ is a strong, but a rather selective oxidiser. Chlorine dioxide prevents the spread of pathogens between animals, from animals to humans and vice versa. ClO_2_ reacts fast with cysteine and methionine (two sulphur‐containing amino acids), with tyrosine and tryptophan (two aromatic amino acids) and with two inorganic ions: Fe^2+^ and Mn^2+^ (Noszticzius et al., 2013).

Due to widespread and continuously emerging antimicrobial resistance, antimicrobial drug consumption is required to be significantly reduced in animal husbandry. Among arising alternative solutions, the hyperpure ClO_2_ (patent: Noszticzius et al., 2007) could be an ideal biocidal additive to the diet of food‐producing animals. It is a great advantage that microbial resistance to chlorine dioxide is unlikely because it acts on the thiol group which is fundamental in all living organisms (Noszticzius et al., 2013). Furthermore, during this pandemic era, the use of safe antiseptics is gaining prominence, as their use has also become generic in everyday life.

Chlorine dioxide has found to be highly biocidal in low concentrations on intestinal biota while simultaneously at this same concentration having no negative effect to daily weight gain (Akamatsu et al., 2012). It is proposed that the animals can safely drink it, without any adverse effect (Ma et al., 2017).

There are no scientific data regarding the biological effects of chlorine dioxide as an intestinal antiseptic in swine. We have chosen the porcine intestinal epithelial cell line (IPEC‐J2) to begin exploring these effects. The gene expression profile of IPEC‐J2 cell cultures makes them suitable for studying the effects of added compounds (Rhoads et al., 1997, Arce et al., 2010, Vergauwen et al., 2015, Razzuoli et al., 2018). The IPEC‐J2 cells are non‐cancerous intestinal columnar epithelial cells that were isolated from neonatal piglet mid‐jejunum (Langerholc et al., 2011). According to these aspects, IPEC‐J2 cell line is an adequate model for preliminary studies investigating the effect of chlorine dioxide.

Our aim is to determine whether chlorine dioxide has any effect on inflammatory markers and cytochrome genes expressed by the small intestinal epithelia. The cytochrome P450 (CYP450) enzymes are involved in drug metabolism, by investigating them we might gain information about the drug interaction properties of this biocidal agent.

## 2 MATERIALS AND METHODS

### 2.1 Cell line and culture conditions

The non‐transformed porcine intestinal epithelial cell line IPEC‐J2, originally isolated from jejunal epithelia of a neonatal unsuckled piglet (Schierack et al., 2006), was a kind gift of Dr. Jody Gookin, Department of Clinical Sciences, College of Veterinary Medicine, North Carolina State University, NC, USA. IPEC‐J2 cells were grown and maintained in complete medium, which consisted of a 1:1 mixture of Dulbecco's modified eagle's medium and Ham's F‐12 Nutrient Mixture (DMEM/F12) (plain medium) supplemented with 5% foetal bovine serum (FBS), 5 μg/ml insulin, 5 μg/ml transferrin, 5 ng/ml selenium, 5 ng/ml epidermal growth factor and 1% penicillin–streptomycin (all from Lonza Group Ltd, Belgium). Cells were grown at 37°C in a humidified atmosphere of 5% CO_2_.

IPEC‐J2 cells were seeded onto six‐well plates (Corning Inc., Corning, NY, USA), coated with 8 μg/cm^2^ rat tail collagen type I (Sigma–Aldrich, Steinheim, Germany), at a density of 10^6^ cells/ml; the volume of complete medium was 2.5 ml. Cells could adhere for 24 h before being washed and re‐fed every other day.

### 2.2 Cell viability test

Influence of chlorine dioxide on the viability of enterocytes was tested. A twofold serial dilution of hyperpure chlorine dioxide solution (Solvocid^®^ Vet, Solumium Ltd, Hungary) was prepared across 7 points in phosphate‐buffered saline (PBS) from 300 to 4.7 ppm. IPEC‐J2 cells were seeded onto a 96‐well plate and incubated with the test substances for 15 min at 37°C in 5% CO_2_. After treatment, the cells were washed two times with PBS and were placed back to the thermostat in complete medium. Viability of IPEC‐J2 cells was measured 24 h after treatment by Neutral red uptake assay as described by Repetto et al. (2008).

### 2.3 Treatment of cell cultures

Before treatment, confluent monolayers of the IPEC‐J2 cells were washed with plain medium. Chlorine dioxide (ClO_2_) dilutions were freshly prepared prior to the experiment. Starting from 300 ppm (4.44 mM) initial ClO_2_ concentration, a six‐membered twofold serial dilution (2 × –64 ×) was made in PBS. Control wells received PBS for the same time period. After 15 min treatment at 37°C in 5% CO_2_, the cells were washed with plain medium and cultured at 37°C in 5% CO_2_ for additional 1 h for PCR studies.

### 2.4 Quantitative PCR measurements

One hour after the treatment, culture medium was removed, cells were collected, mRNA was extracted and cDNA was synthetised according to Palócz et al. (2016). Tested genes of interest were *IL6*, *CXCL8/IL8*, *TNF*, *PTGS (COX2)*, *CAT*, *CYP1A1*, *CYP1A2*, *CYP3A29* and *HSPA6* (Hsp70). Hypoxanthine phosphoribosyl transferase (*HPRT*) and peptidylprolyl isomerase A (*PPIA*) were used as reference genes. Primer sequences are listed in Table 1. Quantitative PCR was performed using the iQ SYBR Green Supermix kit (BioRad, Hercules, CA, USA) on the MiniOpticon System (BioRad) according to Palócz et al. (2019).

**TABLE 1:** Sequence of primer sets for porcine genes, used for quantitative PCR

Gene symbol	Accession number	Primer sequences (5′–3′)	Product size (bp)	Efficiency	Reference
*CXCL8*	NM_213867	F: AGAGGTCTGCCTGGACCCCA R: GGGAGCCACGGAGAATGGGT	126	1.972	Paszti‐Gere et al., 2012
*IL6*	NM_214399	F: TTCACCTCTCCGGACAAAAC R: TCTGCCAGTACCTCCTTGCT	122	1.970	Sakumoto et al., 2006
*TNF*	NM_214022	F: TTCCAGCTGGCCCCTTGAGC R: GAGGGCATTGGCATACCCAC	146	1.873	Hyland et al., 2006
*PTGS2*	NM_214321	F: AGAAGCGAGGACCAGCTTTC R: AAAGCGGAGGTGTTCAGGAG	215	1.905	Farkas et al., 2015
*CAT*	NM_214301	F: CAGCTTTAGTGCTCCCGAAC R: AGATGACCCGCAATGTTCTC	180	1.944	Luci et al., 2007
*CYP1A1*	NM_214412	F: CAGAGCTGCTTAGCCTTATCAACC R: CTGGATGCTGGGATTTGTCACCAG	386	2.00	Kojima et al., 2010
*CYP1A2*	NM_001159614	F: GTGAGGAGATGTTCAGCATCGTGAAG R: CTTCTGTATCTCAGGATATGTCACA	386	1.750	Kojima et al., 2008
*CYP3A29*	NM_214423	F: TTCGTGCTTCACAGAGAGACCC R: TACTAGGTGGGGGTGGATGG	576	1.975	Farkas et al., 2014
*HSPA6*	NM_001123127	F: GCCCTGAATCCGCAGAATA R: TCCCCACGGTAGGAAACG	152	2.0	Zhong et al., 2010
*PPIA*	NM_214353	F: GCGTCTCCTTCGAGCTGTT R: CCATTATGGCGTGTGAAGTC	160	1.907	Hyland et al., 2006
*HPRT*	NM_001032376	F: GGACTTGAATCATGTTTGTG R: CAGATGTTTCCAAACTCAAC	91	1.963	Nygard et al., 2007

Abbreviations: 1A1, family 1 subfamily A member 1; 1A2, family 1 subfamily A member 2; 3A29, family 3 subfamily A member 29; CAT, catalase; CXCL8, C‐X‐C motif chemokine ligand 8; CYP, cytochrome P450; F, forward; HPRT1, hypoxanthine phosphoribosyl transferase 1; HSPA6, heat shock protein family A (Hsp70) member 6; IL6, interleukin 6; PPIA, peptidylprolyl isomerase A; PTGS2, prostaglandin‐endoperoxide synthase 2; R, reverse; TNF, tumour necrosis factor.

### 2.5 Statistical analyses

Relative gene expression levels of the genes of interest were calculated by the Relative Expression Software Tool (REST) 2009 Software which uses the Pair Wise Fixed Reallocation Randomisation Test^©^. Statistical analyses were performed by R version 3.6.3 (R Core Team, 2012; R: A language and environment for statistical computing [R Foundation for Statistical Computing, Vienna, Austria; ISBN 3‐900051‐07‐0]). Differences between means were evaluated by one‐way analysis of variance (ANOVA) followed by a post hoc comparison using Tukey's ‘Honest Significant Difference’ method. Differences were considered significant if the *p*‐value was < 0.05.

## 3 RESULTS

### 3.1 Viability of IPEC‐J2 cells

**FIGURE 1:**
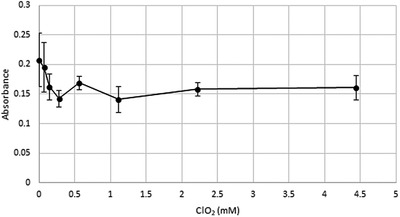
Viability of porcine jejunal cells (IPEC‐J2) after 15 min of chlorine dioxide (ClO_2_) treatment. Data expressed as mean ± SD, *n* = 8/group. The chlorine dioxide was diluted in phosphate‐buffered saline; the 0 mM concentration is the control treatment, contains only phosphate‐buffered saline

Viability of the cells was monitored after 15 min treatment with 0.07–4.44 mM chlorine dioxide (Figure 1). Control cells were incubated for 15 min in PBS. Treatment with different concentrations of chlorine dioxide for 15 minutes did not damage the cells. Compared to the 15‐min PBS treatment, the chlorine dioxide treatment did not decrease the viability of the IPEC‐J2 cells.

### 3.2 Effect of chlorine dioxide on relative expression of inflammatory genes

**FIGURE 2:**
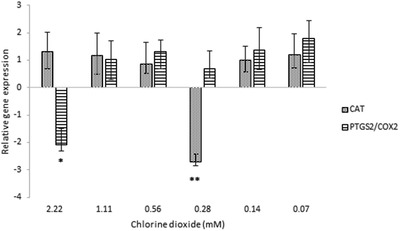
The relative gene expressions of *CAT* and *PTGS2/COX2* at various ClO_2_ concentrations from 0.07 to 2.22 mM in porcine jejunal cell cultures. Results are expressed as mean mRNA expression ratio relative to controls (*n* = 6/group). Significant differences are shown in comparison to untreated controls (*p < 0.05, **p < 0.01). Data are shown as means ± SD. CAT, catalase; PTGS2, prostaglandin‐endoperoxide synthase 2, also known as cyclooxygenase 2 (COX2)

**FIGURE 3:**
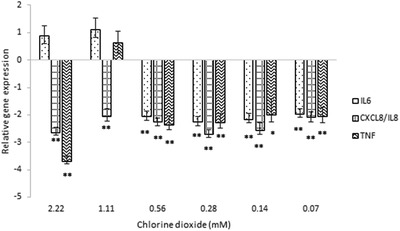
The relative gene expressions of *IL6*, *CXCL8/IL8* and *TNF* at various ClO_2_ concentrations from 0.07 to 2.22 mM in porcine jejunal cell cultures. Results are expressed as mean mRNA expression ratio relative to controls (*n* = 6/group). Significant differences are shown in comparison to untreated controls (*p < 0.05, **p < 0.01). Data are shown as means ± SD. CXCL8, C‐X‐C motif chemokine ligand 8; IL, interleukin; TNF, tumour necrosis factor

**FIGURE 4:**
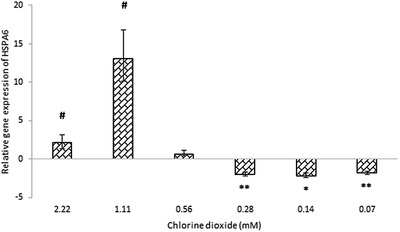
The relative gene expression of *HSPA6* at various ClO_2_ concentrations from 0.07 to 2.22 mM in porcine jejunal cell cultures. Results are expressed as mean mRNA expression ratio relative to controls (*n* = 6/group). Significant differences are shown in comparison to untreated controls (* and # *p* < 0.05, **p < 0.01). Data are shown as means ± SD. HSPA6, heat shock protein family A (Hsp70) member 6

The gene expression of *PTGS2* and *CAT* were not altered by most concentrations of ClO_2_. The 0.28 mM (18.75 ppm) ClO_2_ decreased the level of *CAT* mRNA, and the most concentrated, 2.22 mM (150 ppm) ClO_2_ downregulated the *PTGS2* gene (Figure 2). The expression of *CXCL8* gene was reduced by all concentration of ClO_2_ (Figure 3). *TNF* mRNA was also alleviated by most ClO_2_ concentration except the 1.11 mM (75 ppm). *IL6* gene expression remained unchanged due to the higher concentrations of ClO_2_ – 2.22 and 1.11 mM (150 and 75 ppm) – but it was attenuated by all the other concentrations – 0.07–0.56 mM (4.69–37.5 ppm) (Figure 3). The ClO_2_ treatment had opposite effect on the *HSPA6* (Hsp70) gene: the higher concentrations – 2.22 and 1.11 mM (150 and 75 ppm) – increased the gene expression, and the lower concentrations – 0.28, 0.14 and 0.07 mM (18.75, 9.38, and 4.69 ppm, respectively) – decreased the gene expression (Figure 4).

### 3.3 Effect of chlorine dioxide on relative expression of CYP450 genes

**FIGURE 5:**
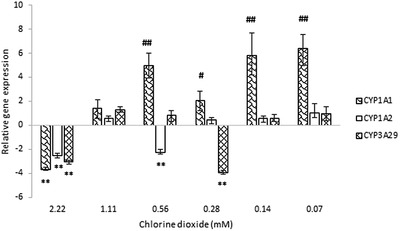
The relative gene expressions of *CYP1A1*, *CYP1A2* and *CYP3A29* at various ClO_2_ concentrations from 0.07 to 2.22 mM in porcine jejunal cell cultures. Results are expressed as mean mRNA expression ratio relative to controls (*n* = 6/group). Significant differences are shown in comparison to untreated controls (# *p* < 0.05, ** and ## *p* < 0.01). Data are shown as means ± SD. CYP, cytochrome P450

The highest ClO_2_ concentration – 2.22 mM (150 ppm) – reduced the expression of all the three investigated CYP genes (Figure 5). Furthermore, the gene expression of *CYP1A2* was decreased by 0.56 mM (37.5 ppm) ClO_2_ and the *CYP3A29* gene was downregulated by 0.28 mM (18.75 ppm) ClO_2_. The *CYP1A1* mRNA level was enhanced after incubation with the lower concentrations of ClO_2_: 0.07–0.56 mM (4.69–37.5 ppm).

## 4 DISCUSSION

Based on the mechanism of action of chlorine dioxide, we hypothesise that negligible amount would reach the small intestine when administered via feed. However, via drinking water administration a significant portion of the administered amount could pass through the stomach if empty, and the incidence of this is increased by long‐term use or inadequate inclusion ratio.

The examined inflammatory and cytochrome genes expressed by the non‐cancerous intestinal epithelial cells were influenced by certain concentrations of chlorine dioxide. The lower concentrations applied decreased the gene expression of *HSPA6*. Heat shock protein 70 is crucial in cell survival: it can prevent apoptosis, repair damaged proteins (Murphy, 2013) and reduce mitochondrial and cellular ROS production (Li et al., 2018).

TNF induces apoptosis (Guicciardi et al., 2000) and also damages the epithelial barrier integrity and stimulate the inflammatory process, inhibition of TNF decreases the production of proinflammatory mediators, reduces the proapoptotic markers and the ileal paracellular permeability (Halpern et al., 2006). Overall, downregulation of TNF can hinder acute or chronic inflammation and tissue necrosis.

IL6 is an acute phase immune mediator that cooperates with host defence when infections or injuries occur. However, permanent presence of IL6 leads to chronic inflammation and the development of other immune‐mediated diseases (Tanaka & Kishimoto, 2014). IL8/CXCL8 is a chemokine and a chemoattractant for neutrophils, neutral killer cells, T‐cells, basophils, and eosinophils (Akdis et al., 2011). CXCL8 is responsible for a faster, more effective and targeted inflammatory response at the site of invasion. Inhibition of CXCL8 production is not favourable during the nursing and fattening period. However, it was shown by Razzuoli et al. (2017) that reduction of IL8 was accompanied by a significant decrease in the ability of *Salmonella* Typhimurium to penetrate IPEC‐J2. Overall, signs of inflammation, such as cytokine production, promote colonisation of *Salmonella* and progression of *Salmonella* infection in piglets (Chirullo et al., 2015). According to this, decreased level of inflammatory mediators – interleukins and chemokines – can be beneficial in the prevention of bacterial colonisation and invasion of the gastrointestinal system. Based on our results, lower concentrations of chlorine dioxide are more likely to cause attenuation in the pro‐inflammatory and inflammatory gene expression.

Only the highest applied ClO_2_ concentration inhibited the gene expression of *PTGS2*, formerly known as cyclooxygenase‐2 (*COX2*), which might cause dramatic decrease in the intestinal level of prostaglandin E2, and as a consequence it would facilitate intestinal inflammation (Tanaka et al., 2009).

The 0.28 mM ClO_2_ concentration decreased the *CAT* mRNA level. Inhibition of catalase will directly result in increased production of reactive oxygen species, which consequently leads to higher apoptosis rate (Majumder et al., 2017).

Concerning drug metabolism, the 1.11 mM ClO_2_ concentration exerted no effect on the transcription of the three investigated *CYP* genes of porcine intestinal cells. The lower ClO_2_ concentrations (0.07 and 0.14 mM) resulted in increased expression of the *CYP1A1* gene; this six‐ to eight‐time increase might result in elevated protein levels which would lead to altered metabolism of the known CYP1A1 substrate drugs such as azole antifungals (Velík et al., 2004) and quinolone antimicrobials (Li et al., 2018). Finding the recommended oral dose that has no effect on the xenobiotic metabolising enzymes would be crucial to avoid drug–feed interactions or any alteration in drug biotransformation.

There were very few studies investigating the effect of per os chlorine dioxide in food‐producing animals; one study demonstrated how 0.4 and 0.5 ppm chlorine dioxide oral treatment for 28 days decreased the occurrence of pathogenic microorganisms such as *Escherichia coli* and *Salmonella* in the intestinal tract while not negatively impacting the daily weight gain and feed palatability of broiler chickens (Sultan et al., 2015). In another study, the broiler feed was supplemented with ClO_2_ powder at 500 and 1000 ppm for 35 days; consequently, the number of *E. coli* was reduced in the ileum and cecum (Ahmed et al., 2015). These results make chlorine dioxide a promising antiseptic agent for oral use. The introduction of the use of chlorine dioxide in pig diets could also be effective in preventing the colonisation and multiplication of pathogens.

According to the outcome of our in vitro studies on the non‐cancerous IPEC‐J2 cells, chlorine dioxide modulated the transcription of the investigated inflammatory markers. The effect of chlorine dioxide on inflammatory markers and drug metabolising enzymes should be supported by in vivo studies and the determination of the appropriate oral concentration remains to be elucidated.

## AUTHOR CONTRIBUTIONS

Emma Bradley contributed in the investigation and methodology. György Csikó contributed in conceptualisation, investigation, methodology, resources, supervision and reviewing and editing. Kristóf Kály‐Kullai contributed in investigation and writing the manuscript. Zoltán Noszticzius contributed in conceptualisation, validation and reviewing of the manuscript. Orsolya Palócz contributed in data curation, investigation, methodology and project administration and drafted the original manuscript.

## CONFLICT OF INTEREST

The authors declare no conflict of interest.

## ETHICS

The authors confirm that the ethical policies of the journal, as noted on the journal's author guidelines page, have been adhered to. No ethical approval was required as no live animal experiments were performed during this study.

### PEER REVIEW

The peer review history for this article is available at https://publons.com/publon/10.1002/vms3.658.

## Data Availability

The data that support the findings of this study are available from the corresponding author upon reasonable request.
